# Longitudinal speech and gross motor function development in children and adolescents with cerebral palsy

**DOI:** 10.1111/dmcn.70300

**Published:** 2026-05-15

**Authors:** Sydney A. Jensen, Katherine C. Hustad

**Affiliations:** 1 Department of Communication Sciences and Disorders, University of Wisconsin-Madison, Madison, WI, USA; 2 Waisman Center, University of Wisconsin-Madison, Madison, WI, USA

## Abstract

**Aim:**

To examine longitudinal changes in speech and gross motor function in children with cerebral palsy (CP) between 4 years and 14 years of age using the Viking Speech Scale (VSS) and the Gross Motor Function Classification System (GMFCS).

**Method:**

In this longitudinal observational study, 44 children (26 male, 18 female) with CP were assessed at ages 4 years and 14 years. VSS and GMFCS classifications were analysed at both time points using polychoric correlations with confidence intervals generated using bootstrapping to identify patterns of stability and change.

**Results:**

Classifications were strongly related at both ages. However, more children tended to change VSS levels (43%; 19/44) between 4 years and 14 years than GMFCS levels (34%; 15/44). Very few children demonstrated concurrent changes in both domains.

**Interpretation:**

Changes in VSS levels may reflect protracted speech development in children with CP. Results indicate that while speech and gross motor abilities are strongly related across development, their patterns of change differ descriptively over time. These findings support the idea that children with CP may benefit from ongoing speech intervention throughout childhood and adolescence, regardless of gross motor function.

Children with cerebral palsy (CP) are heterogeneous in their ability profiles,^1–3^ including the body functions and structures that are involved, the severity of involvement, and the functional consequences (activities and participation) of impairment(s).^3^ Motor impairment is a primary defining feature of CP, but children with CP may also have concurrent vision, hearing, and cognitive deficits^4,5^ as well as language, speech, and communication challenges.^1,6–9^ Constellations of abilities can vary greatly. Consequently, classification tools are essential for describing ability profiles using a common language. A range of classification tools have been developed to describe functional abilities in children with CP, including gross motor,^10^ fine motor,^11^ functional communication,^12^ eating and drinking,^13^ and functional speech^14^ domains.

There is a considerable body of literature examining overall communication function, as measured by the Communication Function Classification System^12^ and the Functional Communication Classification System.^15^ The Communication Function Classification System considers an individual’s communication effectiveness as both a sender and receiver of messages, as well as the pace of communication exchanges. In contrast, the Functional Communication Classification System places greater emphasis on the communicator as a ‘producer’, focusing on social, linguistic, operational, and strategic communication competencies. Both measures classify overall communication ability, regardless of modality. While this global approach is essential for characterizing multimodal communication and functional communication abilities, these measures do not differentiate between verbal and nonverbal communication, making it difficult to discern whether an individual’s communication performance is influenced by speech production difficulties, language and cognitive abilities, or the ability to use augmentative and alternative communication effectively. The Viking Speech Scale (VSS)^14,16^ is a communication-related classification tool that focuses specifically on speech in individuals with CP. VSS levels describe speech motor involvement as inferred from intelligibility ratings for children 4 years of age and older. The VSS differs from the Communication Function Classification System and the Functional Communication Classification System in that it does not consider expressive language, sender/receiver communication roles, or multimodal communication, but instead focuses exclusively on speech abilities. As such, the VSS serves as a reasonable proxy for speech motor involvement, with construct validity evidence supporting it as a measure of speech performance specifically.^17^

In the first longitudinal study to investigate developmental changes in VSS ratings, Long et al.^18^ examined 101 children with CP at 4, 6, 8, and 10 years of age. Findings showed that between age 4 years and 10 years, 55% (*n* = 56) of children maintained the same VSS level, while 45% (*n* = 45) showed a decrease in VSS level, reflecting improvement in functional speaking abilities. Change in VSS level over time was closely tied to severity at age 4 years. Specifically, children in the most severe group, level IV, had a high probability of remaining at this level over the course of the study, whereas children in levels II and III were more likely to show improvement with development. Although there was some fluctuation, reflecting both increased and decreased VSS levels at intermediate ages between 4 years and 10 years, all children either stayed at the same VSS level or moved to a level reflecting improved functioning (lower level) between age 4 years and 10 years, the beginning and end points of the study. These findings indicate that many children with CP make improvements in their functional speech abilities up to age 10 years. Results are consistent with other studies of speech development in children with CP, in which developmental trajectories for speech intelligibility extend through middle childhood, beyond what is considered the typical developmental period for their peers.^19,20^ The extent to which children continue to make improvement in speech motor ability after age 10 years is unknown, but the results may have important clinical implications for dysarthria treatment. Further, the extent to which changes in speech motor ability are co-occurring with gross motor changes is also unknown, thus our understanding of whether speech motor and gross motor functional development occur together or on different timeframes is not yet established. The aim of the present study was to investigate the relationship between speech production and gross motor classifications at early and later ages.

The Gross Motor Function Classification System (GMFCS)^10^ is widely used for characterizing gross motor function in children with CP. GMFCS level at younger ages has been shown to be a good predictor of later abilities. For example, Wood and Rosenbaum^21^ found that GMFCS level at 1 to 2 years of age had a positive predictive value of 0.74 for being able to walk at 6 to 12 years of age for children in mild severity groups and a positive predictive value of 0.57 for children in a more severe group. Similarly, Palisano et al.^22^ found that the stability of GMFCS ratings improved with age, with ratings made before age 4 years showing moderate stability (κ = 0.57), and ratings made after age 4 years demonstrated substantial stability (κ = 0.77). Results indicated that younger children tended to make more functional changes than older children. Collectively, research on the GMFCS suggests that classification is reasonably stable at early ages, with increasing stability as children get older, thus making it a reliable tool for predicting long-term gross motor outcomes.^5^

The relationship between speech and gross motor abilities has received some attention in the literature. Cross-sectional studies suggest that there is a clear relationship between VSS and GMFCS classifications. For example, Choi et al.^26^ investigated functional communication profiles in relation to gross motor function, manual ability, and intellectual ability in children with CP between 4 years and 16 years of age. Results showed a moderate correlation between the GMFCS and the VSS. Similarly, Monbaliu et al.^27^ found a strong relationship between VSS and GMFCS classifications in individuals with dyskinetic CP between the ages of 5 years and 22 years. In a study investigating the prediction of communication disorder severity at 5 years of age from characteristics at 2 years for children with CP, Pennington et al.^17^ found that while GMFCS level was highly predictive of later communication abilities, it was not a significant predictor of speech motor abilities, as measured by VSS. These results suggest that while the VSS and GMFCS are highly correlated in cross-sectional studies, this relationship may differ when examining development longitudinally. However, given that this longitudinal study only spanned 3 years, there remains an opportunity to investigate how the relationship between speech and gross motor abilities may change longitudinally in the same children over a longer range of time and the extent to which early gross motor skills may predict later speech motor abilities. Currently, speech abilities cannot be classified until around 4 years of age, but gross motor skills can be classified as early as 2 years. If speech and gross motor classifications are strongly related at young ages and if that relationship is maintained over time, it may be feasible to identify children who will have speech motor difficulties at ages younger than 4 years on the basis of gross motor abilities. Examining how speech and gross motor development progress in children with CP and whether they follow similar or divergent patterns in terms of change and directionality may provide critical insight into the ways in which these two domains, speech motor and gross motor abilities, develop.

The current study extends the work of Long et al.^18^ by examining both speech motor function (VSS) and gross motor function (GMFCS) at ages 4 years and 14 years in the same children, with the goal of quantifying similarities and differences in developmental change between the two motor domains. We addressed the following research questions: (1) What is the relationship between speech and gross motor classifications at age 4 years and again at age 14 years in the same group of children with CP? Does the relationship between speech and gross motor classifications change between the two age points? (2) Are classifications in the speech domain different at age 4 years versus age 14 years, and are classifications in the gross motor domain different at age 4 years versus age 14 years for children with CP? If so, what is the nature and direction of change in each domain?

Based on existing literature, we hypothesized that the relationship between gross motor and speech classification would generally be strong at both ages, given the nature of CP and the established stability of gross motor development.^28,29^ However, based on our previous work, we did expect to see some change in speech classification toward improvement for many children. The literature on gross motor development suggests that fewer children change in their classifications over time; therefore, we expected to observe different patterns in each domain. Specifically, we expected speech classifications to remain the same or decrease (improve) over time, and gross motor classifications to remain stable.^18,26^

## METHOD

This study was approved by the University of Wisconsin-Madison Social and Behavioral Sciences Institutional Review Board (No. 2018–0580). Assent and written informed consent were obtained from and on behalf of all participants.

### Participants

This study included a subset of children from a larger 15-year longitudinal observational study of approximately 140 children with CP from the upper Midwest region of the USA. Inclusion criteria for the larger study were that children: (1) had a primary medical diagnosis of CP, (2) had normal hearing, confirmed by either a formal audiological evaluation or a distortion product otoacoustic emission screening, and (3) were between the ages of 2 years and 4 years at initial enrollment. For the present study, we examined only children who had completed two laboratory-based data collection sessions, one at age 4 years and one at age 14 years. A total of 46 children completed both sessions in the specified age range. Children with co-diagnoses were not excluded from the study. Two children were excluded from the final sample because one had insufficient data to obtain a GMFCS rating, and the other had recently undergone surgery that temporarily affected their GMFCS score, making it not representative of their gross motor abilities. The final sample had 44 children (26 males and 18 females). Summary statistics and demographic information are provided in Table 1.

### Materials and procedures

#### GMFCS ratings

Gross motor function was classified using the GMFCS^10^ or GMFCS Expanded and Revised (GMFCS E&R),^30^ depending on the date of the child’s visit. Children seen before 2011 were classified using the GMFCS, and those after 2011 were classified using the GMFCS E&R. GMFCS classifications used the same descriptors, regardless of the form used: GMFCS level I: walks without limitations; GMFCS level II: walks with limitations; GMFCS level III: walks using a handheld mobility device; GMFCS level IV: self-mobility with limitations, may use powered mobility; and GMFCS level V: transported in a manual wheelchair. Research indicates that absolute agreement between ratings using original GMFCS and GMFCS E&R forms is high (96.8%),^31^ making it appropriate to use both forms in longitudinal research.^28^

The majority of GMFCS or GMFCS E&R ratings in the present study were made by parents as part of standard parent questionnaires at each in-person data collection session. In cases where parent ratings were missing for the 4-year-old or 14-year-old visit, the first author made a clinical rating using the GMFCS E&R form designed for clinicians and healthcare providers.^32^ At the 4-year-old visit, parents or caregivers completed 89% (39/44) of the GMFCS ratings, while the first author made the remaining 11% (5/44) of the GMFCS ratings. At the 14-year-old visit, parents or caregivers completed 93% (41/44) of the GMFCS ratings, while the first author made the remaining 7% (3/44). Studies examining the reliability of ratings made by parents and healthcare providers have found excellent reliability between the two groups when using direct observation^25,33^ and that scores made by either parents or healthcare professionals are suitable for the research setting.^33^

#### VSS ratings

Speech motor function was classified using the VSS.^14^ The VSS is a four-level ordinal scale designed for use by professionals, including speech and language pathologists, and family members to classify speech motor abilities.^34^ The VSS was developed specifically for children and adults with CP aged 4 years and above. Children’s VSS ratings were classified as follows: VSS level I: no speech motor impairment; VSS level II: imprecise speech that is usually understandable to unfamiliar listeners; VSS level III: speech is unclear and not usually understandable to unfamiliar listeners out of context; and VSS level IV: no understandable speech.

Four-year-old VSS ratings for the present study were previously examined by Long et al.^18^ We employed the same procedures to train raters to make VSS classifications for the 14-year-old visits in the present study. VSS ratings were made by research assistants with an academic background in speech and language pathology but no prior familiarity with the children enrolled in this study. Raters were introduced to the VSS and provided a full copy of the VSS rating form,^16^ which outlines descriptions and perceptual characteristics of each level to aid decision-making. Ratings were made based on audio recordings of the child repeating words and sentences from the Test of Children’s Speech Plus,^35^ with utterances ranging in length up to seven words. Additionally, audio recordings from a 10-minute parent–child interaction, where parents or caregivers engaged in a conversation activity, were used for VSS ratings. Raters used headphones while listening to audio samples and were encouraged to listen to recordings as many times as needed to make their ratings. All ratings were made independently, and raters were blinded to the ratings made by other judges of the children at different ages.

#### Reliability

Interrater reliability for VSS ratings was evaluated by having a second research assistant independently rate a subset of the children. As reported by Long et al.,^18^ at the 4-year-old visit, a second rater assessed 30% of the samples. Cohen’s kappa with squared weights demonstrated strong agreement (k = 0.82) for these ratings. The same approach was applied at the 14-year-old visit, with 30% of the samples rated a second time. At this age, Cohen’s kappa with squared weights indicated perfect agreement (k = 1). All kappa statistics were calculated using the irr package in R (R Foundation for Statistical Computing, Vienna, Austria).^36^

We did not obtain reliability data on GMFCS ratings as these were primarily obtained from parents, years before the present study. An extensive body of literature has documented the reliability of the GMFCS^10,21,22,29,37^ and the GMFCS is widely used throughout the world in clinical practice, research, and epidemiological studies of gross motor abilities in CP.^24,29,37–39^

### Statistical analysis

The dependent variables of interest in the present study were GMFCS ratings at 4 years and 14 years and VSS ratings at 4 years and 14 years. Research question 1 examined the relationships between speech motor (VSS) and gross motor (GMFCS) classifications at age 4 years and then again at age 14 years in children with CP. Additionally, we investigated whether the strength of this relationship changed with age.

To assess the association between GMFCS and VSS scores at both time points, we computed polychoric correlations. Polychoric correlation is a statistical method which estimates the association between two unobserved, continuous variables underlying observed ordinal variables using maximum likelihood methods.^40^ This approach is appropriate when the ordinal categories (e.g. GMFCS and VSS levels) are assumed to reflect underlying latent traits such as functional ability or severity. Polychoric correlations were selected for this study to account for these latent constructs that underlie the ordinal classifications used in the VSS and GMFCS scales. In addition to associations between measures, polychoric correlation coefficients were computed within measures (e.g. GMFCS at ages 4 years and 14 years, VSS at ages 4 years and 14 years) to provide context for interpreting associations between the GMFCS and VSS. Polychoric correlation coefficients were interpreted using the following scale: below 0.40 was a weak correlation, 0.40 to 0.49 was a fair correlation, 0.50 to 0.59 was a moderate correlation, 0.60 to 0.69 was a good correlation, and above 0.70 was a high correlation, as described by Hinkle et al.^41^ We used 95% confidence intervals (CIs) to estimate the magnitude of the effect sizes and precision of the polychoric correlation coefficient. CIs were generated using bootstrapping, a nonparametric resampling procedure that repeatedly samples, with replacement, from the observed data set to generate an empirical sampling distribution of a statistic.^42^ In the present study, bootstrapping was set to 2000 resamples, meaning CIs were derived from 2000 resampled data sets to account for potential variability and provide robust estimates of the correlation coefficients. For each reported polychoric correlation, we checked the associated diagnostic test of bivariate normality provided by the polycor R package^43,44^ which can detect severe departures from bivariate normality and found no issues. The second research question examined the extent to which classifications within gross motor function and speech motor function changed between ages 4 years and 14 years for children with CP, along with the nature and direction of change within each domain. We used descriptive statistics to summarize the data and counted how many children changed classifications for speech function and how many changed classifications for gross motor function. We also examined the overlap between changes in speech function and changes in gross motor function. We used contingency tables based on measure (GMFCS, VSS) and age to describe the change in variables over time.

## RESULTS

### Relationship between speech and gross motor classifications

Polychoric correlation coefficients (*r*) and 95% CIs examining the relationship between speech motor and gross motor classifications at ages 4 years and 14 years are reported in Table 2. Polychoric correlation coefficients indicated a positive high association between VSS and GMFCS scores at both 4 years (*r* = 0.767, 95% CI: 0.596–0.918) and 14 years (*r* = 0.856, 95% CI: 0.695–0.963). CIs indicate the range of plausible correlation coefficients spans from moderate to high positive associations at age 4 years and from good to high positive associations at age 14 years, reflecting consistently large association with good precision. Although the correlation was descriptively higher at age 14 years, the estimated difference between age points was statistically unclear (Δ*r* = 0.0890, 95% CI: −0.0868 to 0.271) with the CI spanning from a weak negative to a weak positive difference. Taken together, these findings indicate a strong association between speech motor and gross motor classifications at both ages. As a sensitivity check, we computed the correlations with a coarser rank-based Spearman method, and observed a similar magnitude for the difference in the correlations (age 4 years *r*_s_ = 0.660, 95% CI: 0.489–0.801; age-14 years *r*_s_ = 0.760, 95% CI: 0.579–0.892; Δ*r*_s_ = 0.00994, 95% CI: −0.0715 to 0.284).

### Classification change in speech and gross motor domains

**FIGURE 1:**
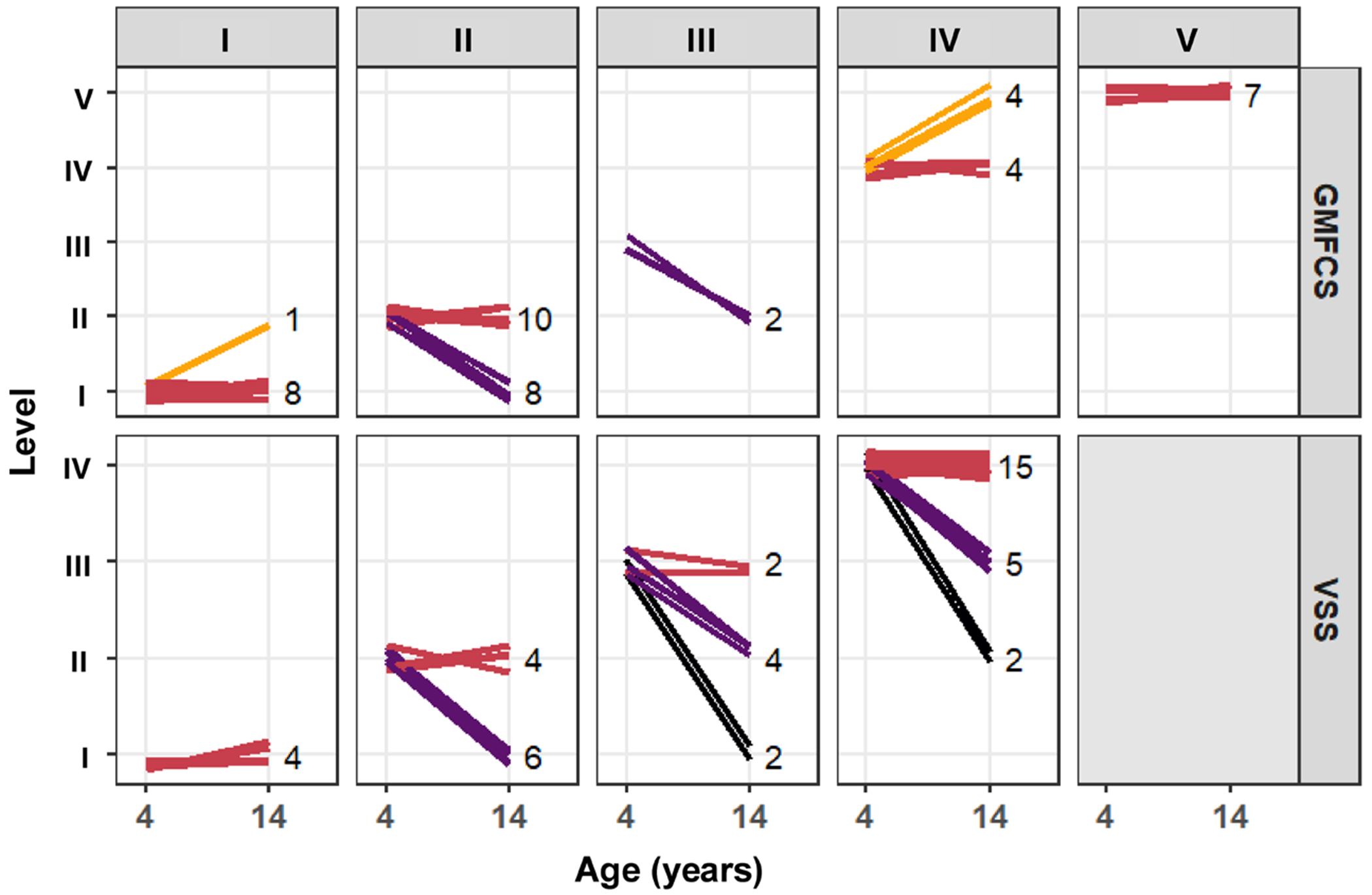
Nature and direction of change in both measures. The *y*-axis shows Gross Motor Function Classification System (GMFCS) and Viking Speech Scale (VSS) levels, while the *x*-axis shows the two age points for each measure. Column headers represent the starting rating on prospective measures at age 4 years. For the VSS level V is grayed out, as level IV is the maximum rating for that scale. Colored lines indicate the change between time points, with pink lines reflecting no change, purple a one-level decrease, black a two-level decrease, and yellow a one-level increase in score.

Figure 1 shows patterns of change in both gross motor classification (top panel) and speech motor classification (bottom panel) between ages 4 years and 14 years. For gross motor abilities, 34% (15/44) of children changed classification levels between 4 years and 14 years. For speech motor abilities, 43% (19/44) of children changed classification levels between 4 years and 14 years. Notably, a slightly larger proportion of children with CP changed in VSS level than in GMFCS level. Polychoric correlation coefficients and 95% CIs, examining the relationship between VSS measures at ages 4 years and 14 years and GMFCS measures at 4 years and 14 years, are provided in Table 2. These correlations reveal similar strong associations between early and late ratings: VSS scores at ages 4 years and 14 years (*r* = 0.948, 95% CI: 0.886–0.989) and GMFCS scores at ages 4 years and 14 years (*r* = 0.970, 95% CI: 0.912–0.993).

Regarding the direction of change, all children who changed their VSS level showed improvement in functional speech motor abilities, as indicated by a decrease in level. Changes in GMFCS level were more variable, with 10 of 15 children having a decrease in GMFCS level (increased functional gross motor abilities), and 5 of 15 children having an increase in GMFCS level (reduced functional gross motor abilities).

Regarding the magnitude of change, of those who changed VSS level, 79% (15/19) improved by one level, while 21% (4/19) improved by two levels. All two-level VSS improvements occurred in children with worse functional speech motor abilities at age 4 years. Between ages 4 years and 14 years, two children changed from a VSS level IV to level II, while two others changed from level III to level I. These changes represent substantial development in functional speech motor abilities. For the GMFCS, all changes were limited to a one-level shift, regardless of whether the change was toward an increase or decrease in GMFCS level.

Only 14% (6/44) of children exhibited concurrent changes in both GMFCS and VSS levels, indicating that the majority of children experienced changes in one domain or the other. Table 3 presents the VSS and GMFCS levels of children who made concurrent changes in speech motor and gross motor function.

## DISCUSSION

This study examined longitudinal changes in speech and gross motor development of 44 children with CP across a 10-year period, offering unprecedented insight into the longitudinal abilities of this population in both domains. Previous work by Long et al.^18^ demonstrated that VSS scores tend to improve in children with CP between the ages of 4 years and 10 years. However, it is unknown how children’s performance on the VSS would compare to the GMFCS, a measure well established as a stable indicator of motor function over time. This study extends results from Long et al.^18^ by providing novel insights into how speech and gross motor trajectories compare over time and describing functional speech abilities in adolescence.

The primary objectives of this study were to examine: (1) the relationship between speech and gross motor development over time and (2) whether classification levels change within each domain between the ages of 4 years and 14 years, and if so, to characterize the direction and nature of those changes. Two key findings emerged. First, VSS and GMFCS scores were clearly and strongly associated across childhood and adolescence. Second, despite this association, the two domains seemed to vary in their developmental trajectories, with differences in the tendency to change across the 10-year span. These findings are further discussed in the following sections.

### Longitudinal association between speech and gross motor abilities

To our knowledge, this is the first study to investigate the relationship between gross and speech motor measures in children with CP in a 10-year longitudinal sample. Positive high associations were observed between VSS and GMFCS scores at both ages 4 years and 14 years, extending previous cross-sectional findings.^33,34^ We found that this relationship was not statistically different between the two age points, suggesting that the relationship between speech motor and gross motor classifications did not change with maturation. This finding is not surprising given the very high correlations between age points for each measure. Findings of the present study are consistent with broader literature documenting associations between gross motor skills and later development across areas of cognition, social skills, and language skills in children with and without neurodevelopmental conditions.^45–48^ Given the earlier timeframe of key gross motor milestones and availability of validated assessments for gross motor function (GMFCS can be administered at age 2 years) compared to speech development (VSS can be administered at age 4 years), observations of gross motor abilities may be a potential indicator of risk for later speech challenges. However, prospective longitudinal studies beginning at early ages using validated fine-grained early speech or oral motor measures are necessary to evaluate this possibility. Preliminary studies by Long and Hustad^49,50^ have begun to explore the extent to which early babbling behavior may be predictive of later speech outcomes; however, studies are needed that tie gross motor developmental milestones together with early vocal behaviors to understand codevelopment across domains.

### Patterns of change in VSS and GMFCS classifications

Consistent with previous longitudinal research,^22,24,41^ GMFCS levels remained relatively consistent, with most children maintaining their same classification from age 4 years to age 14 years; however, 34% of children in the present study showed a change in GMFCS level, which included higher and lower classification levels across children. While most children also maintained the same VSS classification levels over time, 43% of children showed a change in level over the 10-year period; all children showed the same direction of change toward improvement in speech. Generally, our findings are consistent with the literature showing that change in GMFCS classification can be bidirectional, with some individuals showing improvement and others showing decline in gross motor abilities.^21,29^ In contrast, changes in VSS classifications have been shown to be primarily unidirectional, reflecting improvement. This unidirectional trend is likely a reflection of the protracted timeline of speech development in CP, as studies using fine-grained speech assessment have shown that many children with CP are continuing to make refinements in their speech production abilities even through 10 years of age.^18–20^ As such, an improvement in VSS (decrease in level) between ages 4 years and 14 years may be more of a reflection of developmental change and a protracted speech development timeframe than a reduction in speech impairment, per se.

Classification level at 4 years seemed to be associated with whether or not children changed levels at 14 years. This was true for GMFCS level and for VSS level. Children initially classified at intermediate levels (GMFCS levels II–IV; VSS levels II–III) were the most likely to exhibit classification shifts, compared to those at the extreme ends of the classification scales (GMFCS levels I or V; VSS levels I or IV), consistent with previous findings on the GMFCS.^16,19,30^ However, even within the most involved VSS level (IV), nearly half of the children demonstrated an improvement in function (decreased level) at age 14 years. Furthermore, children who were at VSS level IV were also the only participants to experience a two-level change between ages 4 years and 14 years, indicating that even children with the most severe speech involvement in childhood have potential to make gains in their speech production over time. We note an important caveat in this observation related to ceiling and floor effects—children could not get better than a level I nor worse than level IV (on the VSS). Nonetheless, the key point is that the children with the most severe speech involvement were the ones who were most likely to show improvement over time.

Together, these findings highlight the close relationship between functional speech abilities and functional gross motor abilities but also point to nuances that are unique to each domain. While gross motor abilities are considered stable relatively early, speech development in CP often follows a longer time course. By directly comparing change across these systems, this study extends the work of Long et al.,^18^ highlighting subtle differences in patterns of speech and gross motor development in children with CP.

### Implications

While results of the present study are preliminary in nature, they pose considerations for clinical decision-making when using functional classification systems to support diagnosis, goal setting, family education, and prognosis. Although statistically related, VSS and GMFCS classifications changed concurrently in only a small subset of children (13%), and the direction of change was inconsistent. This reinforces the notion that these systems reflect related but distinct developmental constructs, each contributing unique information to a child’s functional profile.

Additionally, the developmental change captured by each measure is fundamentally different: while GMFCS scores are typically stable from age 2 years onward, VSS classifications may be more dynamic, and expected to change, aligning with existing research on speech abilities in children with CP using fine-grained measures.^19,51^ Most notably, this is especially the case with children who have the most severe speech motor impairment. This variability highlights the importance of speech and communication interventions for children with CP, which should be designed to support a prolonged developmental period and remain a priority throughout late childhood and adolescence, regardless of gross motor abilities. Furthermore, it emphasizes the need for a multimodal communication approach, encompassing the use of verbal speech production as well as integration of augmentative and alternative communication modalities to support speech development as well as social participation.

### Limitations and future directions

While findings of this study provide insights into speech abilities in children with CP, several limitations should be considered when interpreting the results. Because of the small number of children in this study, we were not able to account for diagnostic (e.g. CP type, anatomical involvement) or medical (e.g. co-occurring conditions, treatment history) factors that may influence outcomes. In addition, the goal of this study was to examine the relationship between speech and gross motor abilities at two timepoints. However, by exclusively examining data at ages 4 years and 14 years, our findings reflect performance at only two fixed timepoints and do not encompass continuous developmental trajectories across childhood and adolescence. Given the heterogeneous nature of CP, children may have experienced changes in VSS or GMFCS levels at time points before, between, or after the two ages examined in this study.

One potential concern with this study is power. Observed correlations between measures of gross motor and speech function (GMFCS–VSS) and within measures across time (e.g. GMFCS 4–14 years, VSS 4–14 years) all exceeded the threshold of *r* ≥ 0.41, which was the estimated to be the minimum detectable correlation with *n* = 44, α = 0.05, and 80% power. However, more power is required to detect the *difference* between the two GMFVS–VSS correlations. Assuming all other variable correlations are fixed, it would require approximately 21 additional participants to detect the observed difference between correlations of 0.767 and 0.856 (calculation by power.z.twocors.steiger() in the pwrss R package).^52^ As such, it is possible this null result is representative of limited statistical power and should be interpreted with caution.

The unequal distribution of children across VSS levels at age 4 years also made it difficult to interpret proportional data for numbers of children who changed levels. For instance, while 7 of 15 children (47%) classified in VSS level IV improved, a higher proportion of children classified in VSS level III (6/8; 75%) showed improvement. Thus, although more children in VSS level IV improved in absolute number, the proportional change was greater in VSS level III, complicating interpretation of clinical significance across levels. Longitudinal studies that include larger numbers of children are needed to address this issue.

Our study sample was drawn from the upper Midwest region of the USA, which may not reflect the ethnic and socioeconomic diversity of the broad CP population. Additionally, there is a potential selection bias, as the present analyses relied on the presence of complete data, only including children from the greater longitudinal cohort who met the inclusion criteria of having data available at both study timepoints. Children in the original longitudinal cohort did not meet requirement of two session visits for a variety of reasons (e.g. death, relocation) and it is not possible to determine how those with incomplete data may have differed in speech or gross motor function from those included in the present study. Furthermore, families who participated in this longitudinal study may be those with children who have specific speech concerns and are more likely to be active in therapies and resources to support their child’s speech development. As a result, the present sample may represent a subset of children with CP who were healthier, more stable, or had greater access to resources over time, contributing to potential selection bias.

Finally, specific details of treatment to improve speech and gross motor abilities are unknown for the children in the present study. Fine-grained tracking of how therapies may impact functional abilities is necessary in both domains to address this question. At present, the collection of population-level data on individuals with CP is not possible within the US healthcare system. However, efforts to engage service providers to participate in CP registries are gaining traction and hold the potential for large-scale longitudinal studies addressing classification change such as the one presented in this paper.

In the present study, we observed that the VSS, a relatively crude rating of functional speaking ability, can detect refinement of speech abilities in some children with CP. The sensitivity and specificity of change detection by the VSS relative to change characterized using other fine-grained measures^19,51^ is unknown. We also do not know the extent to which change in VSS level reflects meaningful functional change in a child’s speech intelligibility. Future studies are needed to investigate these questions to continue to advance treatment decision-making, optimize participation, quality of life, and educational outcomes for children with CP.

## Conclusion

This study provides new insights into the long-term speech and gross motor development of children with CP, highlighting the strong but complex relationship between these domains. Although statistically associated across development, speech and gross motor ability patterns of change show subtle differences over time. Gross motor classification, as captured by the GMFCS, remains relatively stable from early childhood through adolescence. In contrast, speech motor classification, as measured by the VSS, continues to change well beyond the preschool years, with nearly half of the children showing changes in speech classification between ages 4 years and 14 years. These findings underscore the importance of ongoing speech intervention as well as support for multimodal communication that enables social participation for children with CP. While early gross motor classification may offer some insight into later speech outcomes, variability in developmental trajectories across domains points to the value of considering each construct independently when planning intervention and setting expectations, and continued progress monitoring in speech abilities throughout late childhood and adolescence.

## Figures and Tables

**TABLE 1:** Demographics and clinical characteristics of children with cerebral palsy (*n* = 44).

Variable	*n* (%)
Age group, mean (SD)	
4 years	52 months (2.5 months)
14 years	169 months (6.3 months)

Sex	
Male	26 (59)
Female	18 (41)

Ethnicity	
White	37 (84)
Black	2 (5)
Hispanic/Latino	2 (5)
Asian	1 (2)
Other	2 (5)

CP type^a^	
Spastic	35 (80)
Unknown	4 (9)
Ataxic	4 (9)
Hypotonic	1 (2)

Anatomic involvement	
Unknown	11 (25)
Bilateral (diplegia)	10 (23)
Unilateral—right	8 (18)
Bilateral (quadriplegia)	8 (18)
Unilateral—left	6 (14)
Bilateral (triplegia)	1 (2)

Additional diagnosis	*n*
Vision impairment	22
Seizures	14
Cortical vision impairment	4
Autism	3
ADD/ADHD	4
Anxiety	3
Genetic diagnosis	5
At 4 years only	
Receptive language delay/intellectual impairment	18 (41)

VSS level	*n*
4 years	
I	4
II	10
III	8
IV	22
14 years	
I	12
II	10
III	7
IV	15

GMFCS level	*n*
4 years	
I	9
II	18
III	2
IV	8
V	7
14 years	
I	16
II	13
III	0
IV	4
V	11

a No children in the sample had only dyskinetic CP.

Abbreviations: ADD, attention deficit disorder; ADHD, attention-deficit/hyperactivity disorder; CP, cerebral palsy; GMFCS, Gross Motor Function Classification System; VSS, Viking Speech Scale.

**TABLE 2:** Polychoric correlation coefficients between and within measures of VSS and GMFCS at ages 4 years and 14 years.

Variables	Correlation coefficient	Confidence interval
GMFCS/VSS 4 years	0.767	0.596–0.918
GMFCS/VSS 14 years	0.856	0.695–0.963
Difference between correlations	0.0890	−0.0868 to 0.271
VSS 4 years/VSS 14 years	0.948	0.886–0.989
GMFCS 4 years /GMFCS 14 years	0.970	0.912–0.993
Difference between correlations	−0.0216	−0.0790 to 0.0384

*Note*: 95% confidence intervals were generated with bootstrapping using 2000 resamples. Abbreviations: GMFCS, Gross Motor Function Classification System; VSS, Viking Speech Scale.

**TABLE 3:** Scores and amount of change of the six children who changed both their VSS and GMCS levels.

Participant	VSS 4 years	VSS 14 years	GMFCS 4 years	GMFCS 14 years	Change in VSS	Change in GMFCS
1	III	II	IV	V	1	−1
2	IV	II	I	II	2	−1
3	III	I	II	I	2	1
4	III	I	II	I	2	1
5	III	II	II	I	1	1
6	II	I	II	I	1	1

*Note*: Negative numbers represent an increase in score, with positive numbers representing a decrease in score. Abbreviations: GMFCS, Gross Motor Function Classification System; VSS, Viking Speech Scale.

## Data Availability

The data that support the findings of this study may be made available under limited circumstances and are not publicly available because of privacy or ethical restrictions. Contact Sydney Jensen for more information.

## What this paper adds

- Viking Speech Scale (VSS) and Gross Motor Function Classification System (GMFCS) scores are strongly related across childhood and adolescence.
- More children changed VSS levels than GMFCS levels between 4 years and 14 years of age.
- Very few children made concurrent change in their VSS and GMFCS levels.

## Abbreviation:

VSS: Viking Speech Scale
